# Expression and Functional Properties of an Anti-Triazophos High-Affinity Single-Chain Variable Fragment Antibody with Specific Lambda Light Chain

**DOI:** 10.3390/ijms17060823

**Published:** 2016-06-07

**Authors:** Rui Liu, Xiao Liang, Dandan Xiang, Yirong Guo, Yihua Liu, Guonian Zhu

**Affiliations:** 1 Institute of Pesticide and Environmental Toxicology, Zhejiang University, Hangzhou 310058, China; jianhanjojo@163.com (R.L.); liangxiaozju@126.com (X.L.); xiangdd@zju.edu.cn (D.X.); zhugn@zju.edu.cn (G.Z.); 2 Environment and Plant Protection Institute, Chinese Academy of Tropical Agriculture Sciences, Haikou 571101, China; 3 Research Institute of Subtropical Forestry, Chinese Academy of Forestry, Hangzhou 311400, China

**Keywords:** triazophos, scFv, soluble expression, ELISA, SPR

## Abstract

Triazophos is a widely used organophosphorous insecticide that has potentially adverse effects to organisms. In the present study, a high-affinity single-chain variable fragment (scFv) antibody with specific lambda light chain was developed for residue monitoring. First, the specific variable regions were correctly amplified from a hybridoma cell line 8C10 that secreted monoclonal antibody (mAb) against triazophos. The regions were then assembled as scFv via splicing by overlap extension polymerase chain reaction. Subsequently, the recombinant anti-triazophos scFv-8C10 was successfully expressed in *Escherichia coli* strain HB2151 in soluble form, purified through immobilized metal ion affinity chromatography, and verified via Western blot and peptide mass fingerprinting analyses. Afterward, an indirect competitive enzyme-linked immunosorbent assay was established based on the purified anti-triazophos scFv-8C10 antibody. The assay exhibited properties similar to those based on the parent mAb, with a high sensitivity (IC_50_ of 1.73 ng/mL) to triazophos and no cross reaction for other organophosphorus pesticides; it was reliable in detecting triazophos residues in spiked water samples. Moreover, kinetic measurement using a surface plasmon resonance biosensor indicated that the purified scFv-8C10 antibody had a high affinity of 1.8 × 10^−10^ M and exhibited good binding stability. Results indicated that the recombinant high-affinity scFv-8C10 antibody was an effective detection material that would be promising for monitoring triazophos residues in environment samples.

## 1. Introduction

Organophosphorous pesticides (OPPs), which are known as acetylcholinesterase inhibitors, are widely used in pest control [1]. However, the inappropriate and essentially unregulated use of these compounds has endangered human health because of occupational or environmental exposure [1,2]. Triazophos is a non-systemic broad spectrum OPP that negatively affects organisms. For example, triazophos can enter aquatic environments and cause teratogenicity in fish embryos and larvae [3,4]. In addition, female rats suffer from blood, kidney, and liver toxicities, as well as changes in hormone levels, after long-term exposure to low concentrations of triazophos [5]. Researchers also found that chronic exposure to triazophos significantly impaired the learning and memory function of rats [6]. To minimize the danger of triazophos residues on human health, a strict limit of 0.01 mg/kg in food was set by the British Health and Safety Executive (https://secure.pesticides.gov.uk/MRLs/search.asp). Therefore, regular monitoring of triazophos residues in food and environmental samples is necessary.

Chromatography and mass spectrometry provide excellent accuracy and reliability in determining triazophos residues in different samples [7,8]. However, these methods require time-consuming sample pretreatments, expensive instrumentations, experienced technicians, and lengthy procedures. Acetylcholinesterase inhibition assays are fast but lack specificity because the acetylcholinesterase can be inhibited by OPPs and carbamate pesticides [9]. Immunoassay is a simple, fast, specific, and cost-effective screening technique that offers an alternative to traditional instrumental methods. To date, enzyme-based [10,11] or bead-based [12,13] immunoassay, gold immunochromatographic assay [14], and piezoelectric immunosensors [15] have been developed for triazophos detection. Most of these methods are based on monoclonal antibodies (mAbs) that are specific to triazophos, with the best limit of detection at 0.1 ng/mL. However, mAbs largely depend on hybridoma cell lines, which require specific maintenance procedures for long-term storage [16].

With the rapid development of antibody engineering techniques, recombinant antibodies represent the next generation of mAb. Unlike traditional mAbs, recombinant antibodies can be maintained in bacteria and offer a stable genetic source [16]. Single-chain variable fragment (scFv) is the most popular format of recombinant antibody that has been successfully constructed by assembling the variable-heavy (VH) region and light chain (VL) domain of an antibody with a flexible linker. Compared with mAb, the sensitivity and specificity of scFv to an antigen can be further tailored according to its sequence and can be rapidly immortalized through synthesis and expression [17,18,19]. Apart from scFvs for biomolecules, several functional scFvs against small molecular pollutants have been developed. These include biotoxins such as fumonisin B1 [20], Cry1B toxin [21], and aflatoxin [22]; insecticides, such as parathion [23], chlorpyrifos-ethyl [24], fenitrothion [25], and carbaryl [26]; and fungicides, such as tetraconazole, imazalil, and thiabendazole [27]. A broad specificity recombinant antibody was developed for OPPs, which exhibited a cross reaction to triazophos with IC_50_ of 25.3 ng/mL [28]. However, an engineered scFv, which was not only specific to triazophos but also displayed a high affinity to satisfy the required detection sensitivity, remained lacking.

To date, the particular sequences of immunoglobulin G (IgG) variable regions can be obtained from hybridoma cells that secrete mAb with excellent performance, or selected from commercial libraries via display technology. Compared with non-immunized libraries, the hybridoma cell represents a valuable resource for producing high-affinity recombinant antibodies against their target [21,22]. However, most previously reported hapten-specific scFv antibodies have been developed with a kappa light chain, which requires a complicated screening procedure to avoid interference from a myeloma-derived fusion partner [28,29,30]. In our previous work, a hybridoma cell line 8C10 specific to triazophos was developed, which was identified with an unusual lambda light chain [31]. Thus, this hybridoma cell line was selected as the resource to construct the recombinant scFv antibody in the present work. Followed by correct amplification and assembly, scFv-8C10 was expressed in a prokaryotic expression system in soluble form, evaluated by surface plasmon resonance (SPR) biosensors, and applied in indirect competitive enzyme-linked immunosorbent assay (ic-ELISA). The results showed that this scFv antibody could be used as an effective reagent for triazophos monitoring because of its high affinity to the analyte.

## 2. Results

### 2.1. Cloning the VH and VLλ Genes of mAb-8C10 from Hybridoma

**Figure 1:**
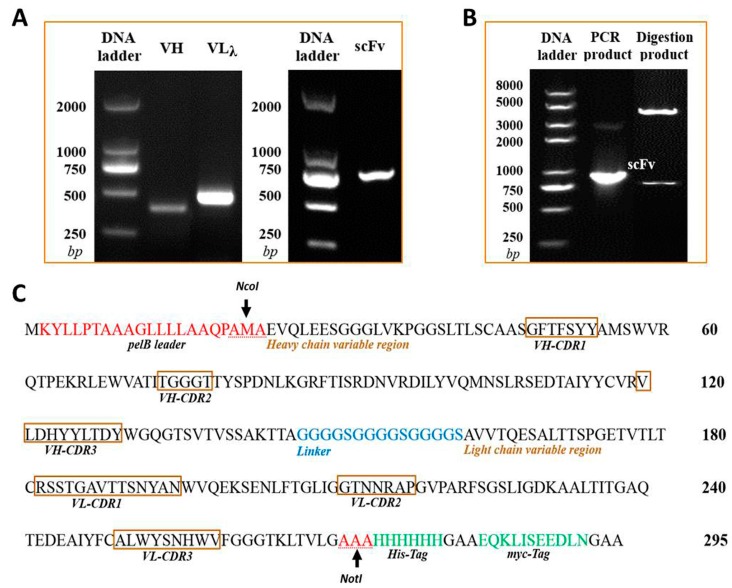
Construction and confirmation of the anti-triazophos scFv-8C10 expression vector. (**A**) The VH and VLλ fragments of anti-triazophos mAb-8C10 gene were amplified and assembled into the scFv-8C10 fragment via SOE-PCR (overlap extension polymerase chain reaction); (**B**) the recombinant expression vector PIT2-scFv-8C10 was confirmed via PCR and restriction enzyme digestion; and (**C**) deduced amino acid sequences of scFv-8C10 expressed in *E. coli* (*Escherichia coli*) HB2151 were according to the nucleotide sequences.

The isotype of murine anti-triazophos mAb-8C10 included IgG1 heavy chain and lambda light chain. With the primer sets of specific isotypes, VH and VLλ genes were amplified from the cDNA of mAb-8C10 (Figure 1A). The sequencing data were compared and validated using the Basic Local Alignment Search Tool of PubMed (http://blast.ncbi.nlm.nih.gov/Blast.cgi). Finally, no aberrant gene was found, and the coding sequences of 372-bp VH and 438-bp VLλ were generated with unabridged functional regions. 

### 2.2. Constructing the Anti-Triazophos scFv-8C10 Fragment and the PIT2-scFv-8C10 Expression Vector

The 737-bp anti-triazophos scFv-8C10 fragment in a VH-linker-VLλ configuration was constructed via splicing by overlap extension polymerase chain reaction (SOE-PCR) (Figure 1A) and confirmed via sequencing. The DNA fragment was digested and ligated into the PIT2 expression vector. As shown in Figure 1B, the scFv-8C10 fragment was amplified via PCR with vector primers and released as an extraneous DNA from the vector after *Nco*I and *Not*I digestion. According to the sequencing data, the scFv-8C10 fragment was constructed as desired and inserted into the correct reading frame of the expression vector.

### 2.3. Expression and Purification of a Soluble Anti-Triazophos scFv-8C10 Antibody

**Figure 2:**
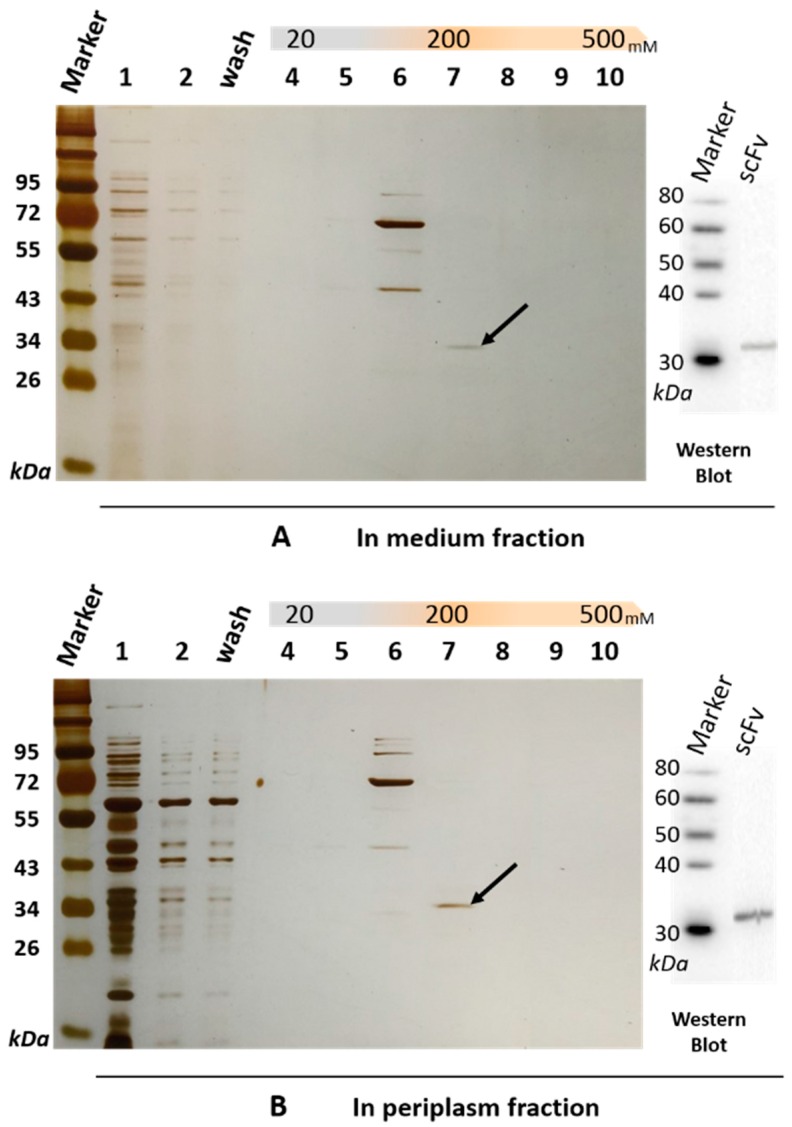
SDS-PAGE and immunoblotting analysis of the soluble anti-triazophos scFv-8C10 antibody purified from the medium fraction (**A**) and the periplasm fraction (**B**) via IMAC (immobilized metal ion affinity chromatography). **Lane 1**, crude protein extract; **Lane 2**, uncombined protein; **Lane 3**, protein washed with binding buffer; **Lanes 4**–**10**, protein eluted with 20, 50, 100, 200, 300, 400, and 500 mM imidazole in a buffer of 20 mM Tris–HCl, pH 7.4, 500 mM NaCl. The scFv-8C10 antibody with a size of 31 kD is pointed out by a black arrow and probed by anti-His-tag antibody on PVDF (polyvinylidene difluoride) membrane.

The soluble scFv-8C10 antibody was expressed in the non-suppressor *Escherichia coli (E. coli)* HB2151 strain. The amino acid sequences of scFv-8C10 expressed in the *E. coli* system were deduced according to the nucleotide sequences, whereas the complementarity-determining regions (CDRs) of VH and VLλ were deduced from the Abysis database (http://www.bioinf.org.uk/abysis/index.html) (Figure 1C). After culture and treatment, a soluble His-fused anti-triazophos scFv-8C10 antibody was expressed and purified via immobilized metal ion affinity chromatography (IMAC). The results from the sodium dodecyl sulfate polyacrylamide gel electrophoresis (SDS-PAGE) showed that a 31 kD protein was eluted down from the medium and the periplasm fraction in the presence of 200 mM imidazole, while some other proteins with molecular weight larger than the theoretic value of target protein were pre-eluted by 100 mM imidazole (Figure 2).

### 2.4. Confirmation of the Anti-Triazophos scFv-8C10 Antibody

Western blot and peptide mass fingerprinting analyses were performed to confirm anti-triazophos scFv-8C10. Anti-His-tag antibody was used as the probe and targeted a 31 kD band on a polyvinylidene difluoride (PVDF) membrane by immunoblotting, either from the medium fraction or from the periplasm fraction (Figure 2). The size was consistent with the bands of the scFv-8C10 antibody on SDS-PAGE (marked by a black arrow in Figure 2), which were sliced and further analyzed via liquid chromatography–electrospray ionization–tandem mass spectrometry (LC–ESI–MS/MS). Table 1 shows the list of all the identified peptides present in the two fractions. Over 99.9% of the fraction sequence identity matched with that predicted of the anti-triazophos scFv-8C10, as accurately identified by MS/MS, because more than two peptides were exactly the same as their functional domains.

### 2.5. Functional Properties of the Anti-Triazophos scFv-8C10 Antibody

**Figure 3:**
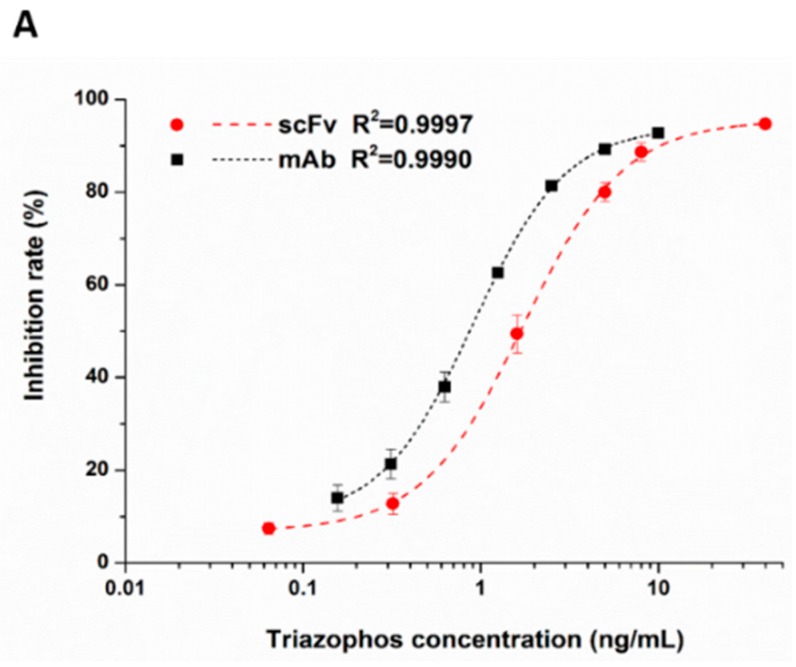

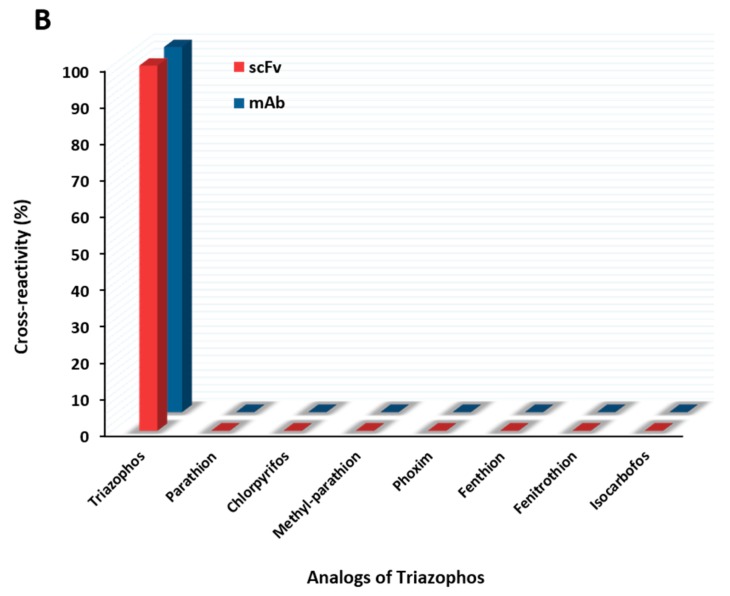
Ic-ELISAs (indirect competitive enzyme-linked immunosorbent assay) for triazophos detection (**A**) and CR of the other analogues (**B**) developed using the purified scFv-8C10 antibody with hapten THBu-OVA conjugate, compared with that of the intact parental antibody. The error bars represent standard deviations calculated from three replicate calibration curves obtained with the same sets of standards. Percentage of CR = [IC_50_ of triazophos/IC_50_ (analogue)] × 100%.

The anti-triazophos scFv-8C10 antibody was characterized via indirect competitive enzyme-linked immunosorbent assay (ic-ELISA). With regard to assay sensitivity, the IC_50_ value of scFv-8C10 to triazophos was 1.73 ng/mL, which was nearly twice as high as that of the intact mAb (0.91 ng/mL), under the condition of the maximum absorbance around 1 (Figure 3A). Furthermore, several analogs of the target triazophos (Supplementary Figure S2) were selected to test cross-reactivity (CR) to the scFv-8C10 antibody. As shown in Figure 3B, the scFv-8C10 antibody exhibited high specificity to triazophos because none of the other analogues was recognized. In addition, unspecific binding was not observed in all the cases of ic-ELISA. These results showed that the scFv-8C10 antibody maintained binding properties similar to the parental mAb, despite a slight reduction in affinity.

### 2.6. Affinity Measurement via Surface Plasmon Resonance (SPR)

**Figure 4:**
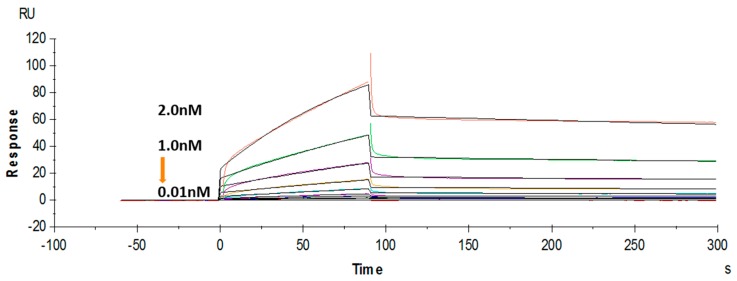
Binding kinetics of the anti-triazophos scFv-8C10 antibody measured via SPR.

The binding affinity of the purified soluble anti-triazophos scFv-8C10 was further tested using a label-free SPR system. Hapten THBu (the functionalized derivative of triazophos) conjugated with ovalbumin (OVA) was coupled to the CM5 sensor chip. Recombinant antibody dose-dependent binding was observed in the THBu-OVA-coated channel, with the association rate constant (*k*_a_) of 9.63 × 10^6^ M^−1^·s^−1^, the dissociation rate constant (*k*_d_) of 1.73 × 10^−3^ s^−1^, and the affinity (*K*_D_) of 1.8 × 10^−10^ M (Figure 4). These results indicated that anti-triazophos scFv-8C10 exhibited extremely strong THBu-binding affinity and stability.

### 2.7. Analysis of Spiked Water Samples

The reliability of scFv-based ic-ELISA was evaluated through recovery experiments in water samples. The results showed that the average recovery values ranged from 84% to 104%, with corresponding inter-assay coefficient of variation (CV) values ranging from 1.2% to 11.8%. Compared with the mAb-based ic-ELISA, these results based on scFv indicate good consistency between the spiking level and the calculated concentration (Table 2). In addition, no false positive was observed in all the cases. Therefore, the purified scFv-8C10 functioned as the anti-triazophos antibody in the immunoassay.

## 3. Discussion

Antibody engineering technology is an effective tool to obtain variable fragments from hybridoma cells that secrete mAb with excellent performance [22]. These fragments do not only focus on immortalizing mAb but are also necessary to generate novel recombinant antibodies (such as scFvs). The scFv antibodies maintained the specific affinity of mAb to antigen and exhibited good application in detecting small molecules, most of which are assembled with a kappa light chain [20,28]. In this work, a murine hybridoma cell line 8C10 that secretes excellent anti-triazophos mAb was chosen as the resource. Consequently, the unabridged VLλ fragment was successfully amplified with the primer set of specific lambda isotypes without the complicated screening procedures of VLκ amplification [29]. This result was possibly related to the less variable lambda gene family reported in [24], as well as the challenge of amplifying functional VLκ from the interference of myeloma-derived fusion partners [30,32]. An aberrant VLκ allele with a premature stop codon was also found in this work (Supplementary Figure S1) if a primer set of a mouse kappa chain was introduced (Supplementary Table S1). This allele was also observed in rat hybridoma, which contained a specific lambda chain and an irrelevant kappa light chain [33]. Therefore, identifying the mAb isotype is necessary. The rare lambda chain probably showed an advantage for functional light chain amplification and could prevent the incorrect amplification of non-functional pseudogenes present in the fusion partners.

A scFv-8C10 specific to triazophos was constructed and expressed in the *E. coli* system based on the successful amplification. In general, a functional recombinant antibody is integrated, soluble, and adopts correct conformations. Enterobacteria *E. coli* is outstanding factory for the expression of recombinant proteins. However, the rapid accumulation of target proteins tends to be misfolded and, thus, these proteins are biologically inactive. In this work, a scFv-8C10 fragment was inserted into the PIT2 vector from Tomlinson library, which comprised an N-terminus *pel*B leader. This leader would direct the protein toward the oxidizing environment of the bacterial periplasm, where disulfide bonds could be formed for proper scFv folding and binding [34,35]. In addition, the expression was mediated with glucose, which could reduce the rate of protein synthesis by repressing the induction of the lac operon with lactose [36]. Finally, the target protein scFv-8C10 was not only expressed in soluble form in the periplasm, but was also secreted into the medium used in this work. Previous reports were based on the PIT2 vector, in which recombinant proteins were only obtained from the periplasm fraction [19,21]. This result was possible because of the existence of periplasmic leader sequences, which may facilitate the protein expressed in periplasm for excretion into the extra-cellular medium [35]. Although the expression amount of the soluble scFv was unsatisfactory, it could be further improved via codon optimization [37,38]. The purified target protein was confirmed via Western blot and peptide mass fingerprinting analyses, and the corresponding results were in agreement with our expectation.

This study aims to obtain a high-affinity triazophos-specific scFv antibody as a potential tool for residue monitoring. Following correct construction and soluble expression, the purified scFv-8C10 antibody was used to develop ic-ELISA to specifically capture free triazophos in solution. The assay sensitivity (IC_50_) was 1.73 ng/mL, comparable to that from the original mAb (IC_50_, 0.91 ng/mL). This engineered anti-triazophos scFv-8C10 antibody was not only highly specific to triazophos, but also satisfied the required detection limit of 0.01 mg/kg. These results are consistent with previous experiences, where high-quality scFv is highly dependent on the property of the hybridoma cell line and can be constructed via homologous recombination of VH or VL gene fragments [22]. Thus, the significantly different binding capability between the anti-triazophos scFv-8C10 and a broad-specific scFv [27] might stem from the different CDRs in variable fragments. Additionally, removing the major framework regions of the parental mAb and adding a linker peptide, which lead to the binding site structure of scFv somehow different from that of the parental mAb, generally, had minimal effect on the recognition capability of the variable regions in our study. This finding agrees with previous reports [27,39,40].

Furthermore, the binding kinetics of anti-triazophos scFv-8C10 and its whole antibody were measured by the SPR immunosensors. The scFv-8C10 presented strong affinity to the immobilized antigen, with approximately *K*_D_ of 1.8 × 10^−10^ M. The fast association rate and slow dissociation rate implied strong binding between the anti-triazophos scFv-8C10 antibody and the coating antigen. However, the sensorgram for mAb showed that the whole antibody came off the immobilized surface extremely slowly and, thus, it was difficult for surface regeneration, which was also observed in a previous report [41]. In comparison to the scFv, this reaction profile of mAb could be due to the formation of stronger bivalent complexes between the mAb and the polyvalent hapten-OVA coated on the chip surface. Anyhow, the newly developed scFv-8C10 antibody is a promising detection reagent compared with other scFvs reported with similar affinities [19,25], which would promote the development of other rapid methods such as lateral flow immunoassays.

## 4. Materials and Methods

### 4.1. Reagents and Materials

Murine hybridoma cell line 8C10 that secreted mAb to triazophos was previously developed in our laboratory. The isotype of mAb was determined using a rapid ELISA mouse mAb isotyping kit from Thermo Scientific (Rockford, IL, USA). Primer sets (Table 3) used to clone the antibody variable genes were synthesized by Shanghai Sunny Biotechnology (Shanghai, China). FastPfu DNA polymerase, dNTPs, pEASY-Blunt Zero vector, and *E. coli* Trans1-T1 competent cells were obtained from Beijing TransGen Biotech (Beijing, China). Restriction enzymes and T4 DNA ligase were from New England Biolabs Inc. (Ipswich, MA, USA). The expression vector PIT2 and the host strain *E. coli* HB2151 were originally from the Tomlinson Library. The media compositions were of biotechnology grade and purchased from Amresco (Solon, OH, USA).

The antigen of hapten THBu (the functionalized derivative of triazophos) conjugated with ovalbumin (OVA) was previously prepared and described in our published research [11]. MaxiSorp™ F96-well polystyrene microplates were obtained from NalgeNunc International (Roskilde, Denmark). The analytical standards of triazophos and other related compounds were purchased from the National Standards Company (Beijing, China). Monoclonal anti-myc-tag antibody and monoclonal anti-his-tag antibody produced in mouse were obtained from Beijing Cw Biotech (Beijing, China). Anti-mouse IgG (whole molecule)-peroxidase antibody produced in rabbit (SecAb-HRP) and 3,3′,5,5′-tetramethylbenzidine (TMB) were supplied by Sigma-Aldrich (Saint Louis, MO, USA). Other reagents were of analytical grade and were obtained from Shanghai Chemical Reagents Company (Shanghai, China).

The buffers used in the ic-ELISA were self-prepared as follows: (i) the coating buffer was 0.05 M carbonate-bicarbonate buffer (CBS, pH 9.6); (ii) the blocking buffer was 0.01 M phosphate-buffered saline (PBS, pH 7.4, 137 mM NaCl) with 2% skimmed milk; (iii) the washing buffer was 0.01 M PBS with 0.05% Tween 20 (PBST, pH 7.4); (iv) the substrate was TMB solution; and (v) the stopping solution was 2 M sulfuric acid.

### 4.2. Cloning of VH and VLλ Genes from Hybridoma

Messenger RNA was purified from total RNA, which was isolated from 5 × 10^6^ hybridoma cells, following the instructions of the manufacturer of the UNIQ-10 Column Trizol Total RNA Isolation Kit and the mRNA purification kit (Sangon Biotech, Shanghai, China). First-strand cDNA was synthesized from mRNA using SuperScriptIII reverse transcriptase (Invitrogen, Carlsbad, CA, USA). The antibody variable genes were amplified from cDNA via PCR using appropriate degenerate oligonucleotide primer sets (see Table 3) with the FastPfu DNA polymerase. The PCR product was analyzed via 1.0% (*w*/*v*) agarose gel electrophoresis and visualized by adding GelRed stains (Biotium, Hayward, CA, USA). A unique band at approximately 400 bp indicated a positive result.

After purification using an agarose gel extraction kit (Takara Biotechnology, Shiga, Japan), the VH and VLλ genes were separately cloned into the pEASY-Blunt Zero vector and then transformed into the *E. coli* strain Trans1-T1 at 42 °C for 30 s. The transformants were selected on Luria Bertani plates supplemented with 50 μg·mL^−1^ ampicillin and kanamycin. After overnight incubation at 37 °C, the plasmids from the culture of colonies were extracted using a mini preparation kit (Axygen, Beijing, China). The positive plasmids were confirmed via PCR and DNA sequencing using the M13 universal primers.

### 4.3. Construction of the Anti-Triazophos scFv-8C10 Fragment and the PIT2-scFv-8C10 Expression Vector

The anti-triazophos scFv-8C10 gene was constructed by assembling the VH and VLλ genes via SOE-PCR. The primers for SOE-PCR are listed in Table 3. First, VH and VLλ were amplified with primers that covered the portion of the linker and the restriction enzyme sites, respectively. Second, the scFv fragment was assembled though the linker from the overlapping genes between the same amount of modified VH and VLλ fragments. The PCR protocol included an initial denaturation at 95 °C for 1 min, followed by 20 cycles of 95 °C for 1 min, 57 °C for 1 min, and 72 °C for 1 min. Using the assembled scFv product as the template, the full-length scFv-8C10 fragment was amplified with *Nco*I-cc-VHF and VLλR-*Not*I primers. After further cloning into the pEASY-Blunt Zero vector and transformation, the positive scFv-8C10 plasmids were produced and identified through the same procedures described above and finally stored at −20 °C.

Afterward, 1 μg plasmid-encoding anti-triazophos scFv-8C10 gene and the PIT2 expression vector were separately digested with *Nco*I and *Not*I restriction enzymes. The digested products of scFv-8C10 fragment and PIT2 vector backbone were purified and verified via agarose gel electrophoresis and ligated with T4 DNA ligation enzyme at 16 °C in a molar ratio of 7:1 to generate the PIT2-scFv-8C10 recombinant vector. The 10 μL ligation products were inoculated into the *E. coli* strain HB2151 competent cells at 42 °C for 60 s and grown with shaking for 1 h at 37 °C. After selection on the plates overnight at 37 °C, the recombinant plasmids from the culture of positive ampicillin-resistant transformants were extracted and evaluated via PCR, restriction enzyme verification, and sequence analysis. Two sequencing primers were used, namely, LMB3 (5′-CAGGAAACAGCTATGAC-3′) and pHENseq (5′-CTATGCGGCCCCATTCA-3′).

### 4.4. Expression and Purification of the Soluble Anti-Triazophos scFv-8C10 Antibody

The expression procedure of the soluble anti-triazophos scFv-8C10 antibody was performed as described in literature [19,21], with a slight modification. The pre-selected positive transformant was incubated in 2 × TY (16 g tryptone, 10 g yeast extract, and 5 g NaCl in 1 L) with 100 mg/mL ampicillin and 1% glucose (2 × TY-A-1%G) at 37 °C with shaking (200 rpm). Then, 1 mL of the overnight culture was transferred into 100 mL fresh medium (2 × TY-A-0.1%G) and incubated at 37 °C with shaking. When the culture reached an optical density (OD) of 0.9 at 600 nm, 1 mM isopropyl-β-d-1-thiogalactopyranosid was added, and the culture was incubated overnight at 30 °C with shaking. The cell cultures were centrifuged at 3300× *g* for 30 min, and the medium fraction was collected and stored at 4 °C. The cell pellet was resuspended in 5 mL 1× TBS buffer (0.2 M Tris–HCl pH 8.0, 0.5 mM EDTA, 0.5 M sucrose), followed by sufficient mixing with 7.5 mL 1/5× TBS buffer (diluted with sterile water). After incubation on ice for 1 h, the suspension was centrifuged at 12,000× *g* for 20 min at 4 °C to obtain the supernatant-containing soluble protein expressed in the periplasm fraction. The product was dialyzed against PBS buffer and stored at 4 °C.

The soluble protein in the medium and the periplasm extracts were concentrated using a 10K MWCO centrifugal concentrator (Millipore, Billerica, MA, USA). The purification of the scFv-8C10 antibody was performed via Ni metal ion affinity chromatography using a 1 mL HisTrap™ HP column (GE Healthcare, Uppsala, Sweden) according to the instructions of the manufacturer. In general, the column was washed with distilled water and equilibrated with a binding buffer (20 mM Tris–HCl, pH 7.4, 500 mM NaCl, 10 mM imidazole) at a flow rate of 1 mL/min. The protein sample pre-diluted with the binding buffer (1:1 volume ratio) was passed through a 0.22 μm filter prior to column loading. Afterward, the column was washed sequentially with the binding buffer and a linear gradient elution buffers (20 mM Tris–HCl, pH 7.4, 500 mM NaCl, and containing imidazole from 20 to 500 mM). For detection, each fraction was collected separately and checked via 12% SDS-PAGE with silver straining.

### 4.5. Protein Confirmation

After separation via SDS-PAGE, the proteins were transferred to an Immuno-Blot PVDF membrane (Bio-Rad, Hercules, CA, USA, 0.2 μm) to identify the purified anti-triazophos scFv-8C10 antibody via immunoblotting, as described in a published method [42]. Furthermore, the band of interest protein was sliced from the SDS gel and verified commercially with ProtTech via LC–ESI–MS/MS. In short, the protein in-gel was digested with sequencing-grade modified trypsin (Promega, Madison, WI, USA) in the digestion buffer (ammonium bicarbonate 100 mM, pH8.5). The dissolved peptide mixture was analyzed via LC–ESI–MS/MS, in which an HPLC system (Agilent, Santa Clara, CA, USA) with a reverse phase C18 column was coupled online with an ion trap mass spectrometer (Thermo Fisher Scientific, Waltham, MS, USA) ionized in an ESI process. The mass spectrometric data were used to search against the target protein sequence of the anti-triazophos scFv-8C10 antibody with ProtTech’s ProtQuest software suite (ProtTech Inc., Philadelphia, PA, USA).

### 4.6. Ic-ELISA

Ic-ELISA for determining the sensitivity of the scFv-8C10 antibody to triazophos was developed. All reactions were carried out at 37 °C. First, 100 μL THBu-OVA diluted in CBS was added into microplate wells and incubated at 37 °C for 2 h. The plates were then saturated with blocking buffer (300 μL/well) for 1 h to minimize the nonspecific interaction signal. Then, 50 μL serially-diluted triazophos standard in PBS and the same volume of the scFv-8C10 antibody (diluted in PBS) were, respectively, added to the wells for 1 h of incubation. The scFv was detected with 100 μL/well anti-myc tag antibody (1:3000 of dilution in blocking buffer) and then probed with 100 μL/well SecAb-HRP (1:40,000 of dilution in blocking buffer) for a two-step 1 h incubation. After each incubation, the plates were washed thrice with washing buffer using a 96-channel washer (Highcreation, Shenzhen, China). Finally, the enzymatic reaction was indicated by the substrate solution (100 μL/well) for 15 min, and then stopped with 2 M sulfuric acid solution (50 μL/well). The absorbance of each well at 450 nm (OD_450nm_) was measured using a SpectraMax i3 microplate reader (Molecular devices, Sunnyvale, CA, USA). All analyses were performed in triplicate. Standard competitive curves were obtained by plotting the inhibition rates against the logarithm of the triazophos concentrations. The data were fitted with the four parameter logistic equations using Origin 8.5 software, by which the IC_50_ values were calculated. Meanwhile, control test without anti-myc tag antibody was checked to eliminate the influence of unspecific binding between SecAb-HRP and scFv-8C10.

Furthermore, the scFv-based ic-ELISA was assessed by identifying triazophos in water samples from different sources, such as tap water (Hangzhou, China), lake water (West Lake in Hangzhou, China), and paddy water (experimental paddy field in Zhuji, China). After filtration, the water samples (pH 7.0–7.4) were fortified with triazophos standard at 1, 2.5, and 5 ng/mL, respectively; the samples were directly analyzed via ic-ELISA without further extraction. Each concentration was replicated thrice to verify repeatability. Meanwhile, control samples without triazophos were tested via immunoassay to eliminate the influence of false positives.

### 4.7. SPR Measurement

The binding activity of the anti-triazophos scFv-8C10 antibody to THBu-OVA was measured using Biacore T200 instrument (GE Healthcare). THBu-OVA and the free OVA were separately immobilized on the CM5 chip surface (flow cell 2 and 1) via the amine coupling method, with the immobilization level of 3123 RU and 3414 RU, respectively. The antibody was diluted with running buffer (10 mM PBS, 137 mM NaCl, 2.7 mM KCl, and 0.05% (*v*/*v*) surfactant P20, pH 7.4) to the corresponding concentration and allowed to flow through the immobilized chip for 90 s 30 μL/min. The antibody was bound to THBu-OVA and then dissociated for 600 s. The chip surface was regenerated between binding cycles with 30 s injection of 10 mM NaOH at 10 μL/min. The sensorgram obtained for the OVA flow cell was subtracted to correct the nonspecific binding and bulk signal from the solution. Data were analyzed using BIA evaluation 3.0 software with 1:1 fit model.

## 5. Conclusions

To summarize, anti-triazophos scFv-8C10 was correctly constructed in this study, and could be rapidly immortalized through synthesis and expression, with no need of animal sacrifice for antibody production. Similar to the mAb-8C10-based ic-ELISA, the scFv-8C10-based ELISA was not only highly specific to triazophos, but it also satisfied the required detection sensitivity of triazophos residues in the water samples. Kinetic affinity evaluation showed that scFv-8C10 displayed high and stable binding to the coating antigen. Therefore, the scFv-8C10 antibody can be a good alternative for the parental mAb and will serve as an effective material to further develop various immunoassays. Moreover, the developed immunoreagent can also provide a basis for engineering multivalent or multifunctional formats of recombinant antibodies for monitoring triazophos residues in the environment and in food samples.

## Figures and Tables

**Table 1:** Identification of the purified anti-triazophos scFv-8C10 antibody from the medium and periplasm fractions via peptide mass fingerprinting.

Peptide No.	Medium Fraction	Periplasm Fraction	Regions
1	LEWVATITGGGTTYSPDNLKGR	LEWVATITGGGTTYSPDNLKGR	VH-FR2-CDR2
2	DILYVQMNSLR	DILYVQMNSLR	VH-FR3
3	VLDHYYLTDYWGQGTSVTVSSAK	– ^1^	VH-CDR3-FR4
4	SSTGAVTTSNYANWVQEK	SSTGAVTTSNYANWVQEK	VLλ-CDR1-FR2
5	SENLFTGLIGGTNNR	SENLFTGLIGGTNNR	VLλ-FR2-CDR2
6	FSGSLIGDK	FSGSLIGDK	VLλ-FR3
7	– ^1^	LTVLGAAAHHHHHHGAAEQK	VLλ-FR4-His-tag
8	LISEEDLNGAA	LISEEDLNGAA	myc-tag

^1^ indicates undetectable.

**Table 2:** Recoveries and precision of triazophos in spiked water samples determined by scFv-based ic-ELISA and mAb-based ic-ELISA (*n* = 3).

Sample	Triazophos Added (ng/mL)	scFv-Based ic-ELISA		mAb-Based ic-ELISA	
		Recovery (%)	CV (%)	Recovery (%)	CV (%)
Tap water	1.0	90	8.8	96	6.8
	2.5	84	5.2	94	8.4
	5.0	90	4.4	86	8.0
Lake water	1.0	110	4.5	98	7.2
	2.5	104	3.1	105	5.3
	5.0	100	1.2	92	6.9
Paddy water	1.0	90	10.0	94	10.6
	2.5	88	11.8	91	7.5
	5.0	104	8.3	85	10.2
Mean		95.6	6.4	93.4	7.9

**Table 3:** Primer sequences for the variable fragments and the full-length anti-triazophos scFv-8C10 gene amplification.

Name	Sequence (5′-3′)	Annotation
*VHF*	SARGTNMAGCTGSAGSAGTC	IgG1 [43]
*VHR*	TGGGGSTGTYGTTTTGGCTGMRGAGACRGTGA	
*VLλF*	GGGAATTCATGGCCTGGAYTYCWCTYWTMYTCT	Lambda (Novagen Ig-primer sets)
*VLλR*	CCCAAGCTTAGCTCYTCWGWGGAIGGYGGRAA	
*NcoI-cc-VHF*	CCATGGCCGAAGTGCAGCTGGAGGAGTC	Anti-triazophos scFv-8C10 fragment
*VHR-Linker*	GCCAGAGCCACCTCCGCCTGAACCGCCTCCACCGGCTGTTGTTTTGGCTGAAG	
*Linker-VLλF*	TCAGGCGGAGGTGGCTCTGGCGGTGGCGGATCGGCTGTTGTGACTCAGGAATC	
*VLλR-NotI*	GCGGCCGCGCCTAGGACAGTCAGTTTG	

*Nco*I, *Not*I sites are underlined; the Linker sequence is represented by being shadowed.

## Supplementary Materials

Supplementary materials can be found at http://www.mdpi.com/1422-0067/17/6/823/s1.

## Abbreviations

The following abbreviations are used in this manuscript:

OPPs	Organophosphorous pesticides
IgG	immunoglobulin G
mAb	monoclonal antibody
scFv	single-chain variable fragment
VH, VL	variable heavy region, variable light region
CDRs	complementarity-determining regions
SOE-PCR	splicing by overlap extension polymerase chain reaction
IMAC	immobilized metal ion affinity chromatography
ic-ELISA	indirect competitive enzyme-linked immunosorbent assay
SPR	surface plasmon resonance
CV	coefficient of variation
