# Supplementary material for: Expression and Functional Properties of an Anti-Triazophos High-Affinity Single-Chain Variable Fragment Antibody with Specific Lambda Light Chain
Source: Int J Mol Sci. 2016 Jun 7;17(6):823. doi: 10.3390/ijms17060823 (PMC4926357; doi:10.3390/ijms17060823)
Supplement: Supplementary file 1 [file ijms-17-00823-s001.pdf]

Supplementary Materials: Expression and Functional Properties of an Anti-Triazophos High-Affinity Single-Chain Variable Fragment Antibody with Specific Lambda Light Chain

Rui Liu, Xiao Liang, Dandan Xiang, Yirong Guo, Yihua Liu and Guonian Zhu

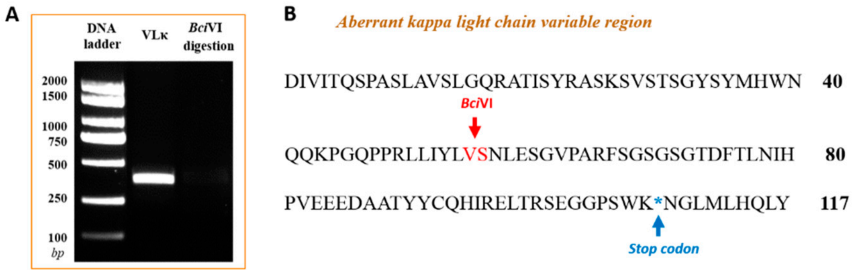

Figure S1. Amplification of the variable gene with primer set for the kappa light chain. (**A**) The aberrant VLκ fragment of the anti-triazophos mAb-8C10 gene was amplified and could be digested by the *Bci*VI restriction enzyme [30]; and (**B**) deduced amino acid sequence of an aberrant premature VLκ fragment.

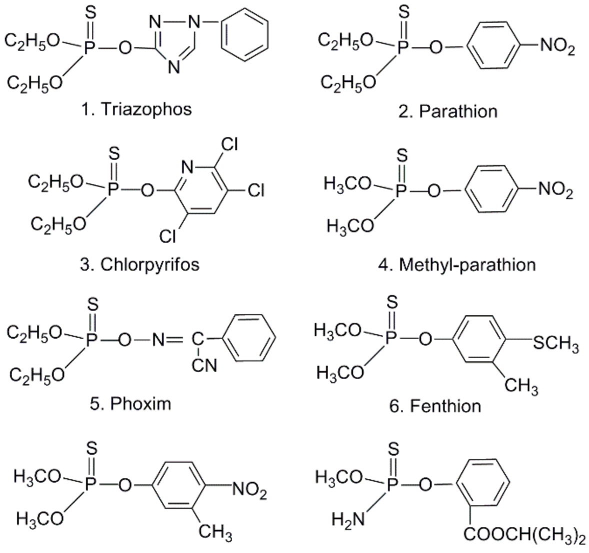

Figure S2. Chemical structures of triazophos and related compounds used in ic-ELISA.

Table S1. The primer set for the amplification of variable fragment of the kappa light chain.

Name	Sequence (5′-3′)	Annotation
VLκF	GAYATTGTGMTSACMCARWCTMCA	kappa light chain [43]
VLκR	GGATACAGTTGGTGCAGCATC	
